# Impact of ISP Tuning on Object Detection

**DOI:** 10.3390/jimaging9120260

**Published:** 2023-11-24

**Authors:** Dara Molloy, Brian Deegan, Darragh Mullins, Enda Ward, Jonathan Horgan, Ciaran Eising, Patrick Denny, Edward Jones, Martin Glavin

**Affiliations:** 1 School of Engineering, University of Galway, University Road, H91 TK33 Galway, Ireland; 2 Ryan Institute, University of Galway, University Road, H91 TK33 Galway, Ireland; 3 Valeo, Tuam, Co., H54 Y276 Galway, Ireland; 4 Department of Electronic and Computer Engineering, University of Limerick, Castletroy, V94 T9PX Limerick, Ireland

**Keywords:** image signal processing, object detection, ADAS, autonomous vehicles

## Abstract

In advanced driver assistance systems (ADAS) or autonomous vehicle research, acquiring semantic information about the surrounding environment generally relies heavily on camera-based object detection. Image signal processors (ISPs) in cameras are generally tuned for human perception. In most cases, ISP parameters are selected subjectively and the resulting image differs depending on the individual who tuned it. While the installation of cameras on cars started as a means of providing a view of the vehicle’s environment to the driver, cameras are increasingly becoming part of safety-critical object detection systems for ADAS. Deep learning-based object detection has become prominent, but the effect of varying the ISP parameters has an unknown performance impact. In this study, we analyze the performance of 14 popular object detection models in the context of changes in the ISP parameters. We consider eight ISP blocks: demosaicing, gamma, denoising, edge enhancement, local tone mapping, saturation, contrast, and hue angle. We investigate two raw datasets, PASCALRAW and a custom raw dataset collected from an advanced driver assistance system (ADAS) perspective. We found that varying from a default ISP degrades the object detection performance and that the models differ in sensitivity to varying ISP parameters. Finally, we propose a novel methodology that increases object detection model robustness via ISP variation data augmentation.

## 1. Introduction

Deep learning-based object detection has substantially outpaced traditional object detection methods [1]. Advances in deep learning (DL) architectures [1], training methods [1], large datasets [2], and hardware accelerators [3] have solidified DL as the dominant technology in this field. Applications such as surveillance [4], manufacturing [5], robotics [6], and even safety-critical applications such as advanced driver assistance systems (ADAS) and autonomous vehicle research [7] are currently utilizing DL models. One drawback of DL object detection is their black-box design. They learn object representations from training data based on a loss function [1]; however, it is unknown how much the appearance of an object can vary before the model can no longer detect it. In contrast, traditional object detectors have hard-coded feature representations of objects [1] that are used to search through the image, making their errors easier to explain. The robustness of multiple object detection architectures to changes in the ISP parameters is characterized in this study.

**Figure 1:**
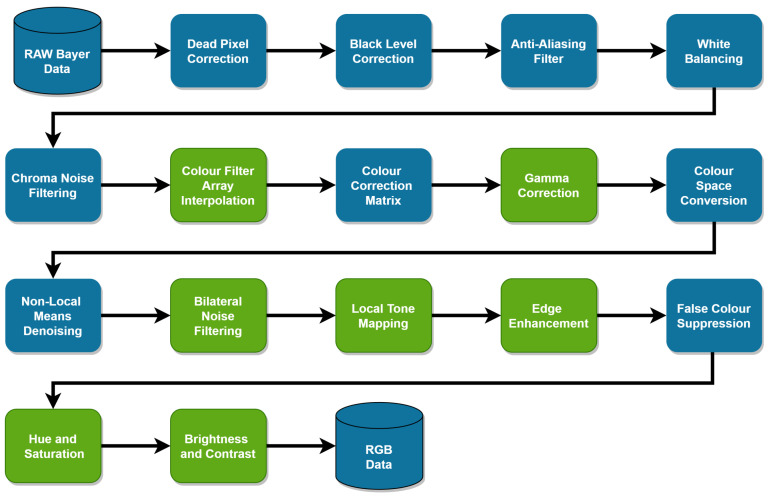
The software ISP pipeline in OpenISP [11,12]. The parameters associated with the green ISP blocks have been varied in this study to evaluate object detection model robustness.

Current public object detection image datasets [8,9] commonly used to develop DL models are generally comprised of images that have undergone image signal processing (ISP) and compression. The ISP turns the brightness data, captured by the sensor’s photodiodes, into red, green, and blue pixel data to be visualized on a display. ISPs are generally tuned subjectively for optimal human perception by experts, often with tens to hundreds of adjustable parameters depending on the features of the ISP. Given that the process involves such a large number of parameters and is subjective, it is highly unlikely that any two ISPs would be identically configured. A typical ISP pipeline is shown in Figure 1. Existing hardware ISPs typically expose a set of registers to the user that are used to set the ISP parameters, with minimal information about the underlying proprietary algorithms that make up the different ISP blocks [10]. Image signal processing can significantly impact the overall appearance of an image; therefore, object detection models trained with post-ISP images are likely to learn features of the object classes coupled with these subjectively tuned ISP parameters. Strategies such as data augmentation are implemented to make learned object representations more generalized and robust. However, the performance impact caused by varying the ISP has not previously been investigated. In this study, we investigate how the commonly utilized methodology of subjective ISP parameter tuning can impact the object detection performance by varying individual ISP parameters and quantifying the impact on performance. Characterizing the relationship between ISP parameters and potentially safety-critical object detection systems such as ADAS algorithms is vital to ensure that system safety is maintained.

In this study, we utilize raw image datasets, without ISP or compression applied, an open-source software ISP [11,12], and a range of object detection models. We benchmark different ISP configurations with a range of object detection models to determine the relationship between ISP and object detection key performance indicators (KPIs). We investigate whether each object detection architecture exhibits a similar performance given the different ISP configurations, taking a detailed look at each algorithm’s errors and prediction confidence levels. We then propose a novel approach for deep learning data augmentation that can make object detection models more robust to deployment on camera systems with different ISP parameters.

This paper is organized as follows: firstly, related works are discussed in Section 2. Section 3 contains the methodology and results for analyzing the performance of multiple object detection algorithms with varying ISP configurations on our original raw dataset. Section 4 outlines our novel data augmentation approach. Section 5 contains the conclusion.

## 2. Related Works

### 2.1. Impact of the ISP in Computer Vision Tasks

The parameters of ISPs have traditionally been tuned subjectively for optimal human vision because, until relatively recently, camera systems have been generally used for human viewing and subjective assessment is the most reliable form of quality measurement [13]. Given that subjective tuning for human vision is the most commonly utilized methodology for ISP tuning, this is referred to as the default ISP configuration.

Camera pipelines are currently being created either solely for computer vision applications or use a dual-ISP design for both human and computer vision. Computer-vision-only camera systems make it possible to directly analyze and tune ISP parameters for specific computer vision KPIs. Buckler et al. [14], in 2017, first investigated how an ISP alters convolutional neural network (CNN)-based algorithms, looking specifically at enabling or disabling ISP blocks using a simplified ISP pipeline. Pseudo-raw images were generated from post-ISP datasets using a reversible ISP model [15] and a sensor noise model [16]. The reverse ISP methodology provided only an approximation, however, and the resulting images lost information that would have been present in the raw data [15]. Artifacts of compression, edge enhancement, and other ISP algorithms were also not considered within the reverse ISP methodology [15]. The study reported that enabling or disabling the demosaicing and the gamma compression ISP blocks yielded the biggest performance degradation. Faster RCNN with the VOC 2017 dataset was utilized and showed a 10% performance degradation when demosaicing or gamma compression was disabled. Yahiaoui et al. [17] followed this research by varying the parameters associated with sharpening and contrast ISP blocks and evaluating the performance on a feature-matching task utilizing post-ISP data, where they found a measurable impact on the feature-matching performance. In 2019, Yahiaoui et al. [18] varied sharpening and contrast ISP blocks on images and investigated the performance impact on feature matching and pedestrian detection. There was a 14% accuracy improvement for the pedestrian detection task by tuning the parameters associated with these ISP blocks specifically for the detection task.

Mosleh et al. [10] introduced a methodology to optimize hardware ISPs using the object detection performance as the optimization function. The covariance matrix adaption evolution strategy (CMA-ES) [19] optimized the ISP parameters to maximize object detection average precision (AP) [9]. Mosleh et al. achieved a 30% AP increase with their optimized ISP over a manually tuned ISP on an automotive object detection task consisting of 440 test images. In 2021, Hansen et al. [20] sought to determine whether ISPs have a performance impact on deep learning models. A reverse ISP method was used to acquire pseudo-raw images of the ImageNet dataset. Training was then carried out on MobileNet classifiers with the original RGB data and with the pseudo-raw data. The model trained with RGB images achieved up to a 12.2% higher accuracy than the model trained on the approximated raw data. Hansen et al. also processed the ImageNet images with a software ISP to benchmark the classification performance against different ISP blocks and found that enabling the tone mapping ISP block increased the model accuracy by 5.8%. Following on the work completed by Mosleh et al. [10], Robidoux et al. [21] presented a methodology to optimize the hardware ISP of HDR cameras using an object detection KPI as the reward function, where they achieved a 33% increase in mAP and mAR over an expert-tuned ISP. They utilized their own raw ADAS dataset and the YOLOv4 [22] object detection algorithm.

In recent years, studies evaluating ISP for computer vision have focused on optimizing high-level object detection KPIs such as AP without investigating whether the distribution of errors has changed due to the changing ISP parameters. There is a need to investigate whether these ISP changes worsen the object detection performance in outlier cases, such as detecting smaller objects. There are no known studies that characterize the object detection performance impact associated with a varying set of ISP parameters on a raw image dataset. There is also a need to investigate whether this impact is consistent across multiple object detection architectures to determine whether some architectures could be more robust or sensitive to changes in the ISP parameters. Many publications make use of a reverse ISP methodology [14,20] that generates pseudo-raw data due to the lack of large-scale raw datasets. These pseudo-raw images provide useful insight into performance trends; however, the use of a truly raw dataset, as is the case with [21], is necessary to accurately represent a real camera system implementation.

### 2.2. Raw Datasets

There is a need for large-scale raw datasets for object detection [23] to analyze the impact of subjective ISP tuning on safety-critical object detection systems and to enable research into tuning ISPs to improve system safety and reliability. The only known publicly available, annotated, and raw object detection dataset is the PASCALRAW dataset [24]. PASCALRAW contains 4259 raw images taken with a Nikon D3200 DSLR camera at a resolution of 24 MP. The dataset has person, car, and bicycle classes with 6550 object instances. The images are 12-bit Nikon raw files that contain the raw Bayer sensor data. The dataset has been annotated at a resolution of 600 × 400 and consists of scenes from Palo Alto and San Francisco during daytime conditions. With just 6550 object instances, PASCALRAW is not large enough to train object detectors from scratch without overfitting, and the distribution of the objects is heavily skewed towards larger objects. Small objects are generally harder to detect, so within the standard object detection benchmark, COCO [9], a metric, was created focusing specifically on objects with a size less than 32 px × 32 px, called APsmall. At the annotation resolution of 600 × 400, none of the objects in the PASCALRAW dataset fall into the COCO APsmall threshold, with the smallest object being 145 px × 145 px. In the COCO dataset, however, 41.4% of the objects fall into the APsmall category, highlighting that the PASCALRAW dataset is heavily skewed toward large objects, which is an easier detection task. Given that PASCALRAW contains 50× fewer object instances and much larger objects than COCO, there is a need for a larger raw image dataset containing smaller objects.

## 3. Evaluating Impact of ISP on Object Detection

### 3.1. Dataset

Many papers that looked into the optimization of ISPs utilized common large-scale post-ISP RGB datasets but processed them with a reverse ISP step as introduced by Buckler et al. [14]. As discussed, ISPs have a large search space and are generally tuned subjectively, which means that the image signal processing cannot be reversed to regenerate the original Bayer data for those images. For this study, we investigate the use of PASCALRAW [24] and our original raw dataset. The details of our dataset are outlined below.

#### 3.1.1. Original Dataset

**Figure 2:**
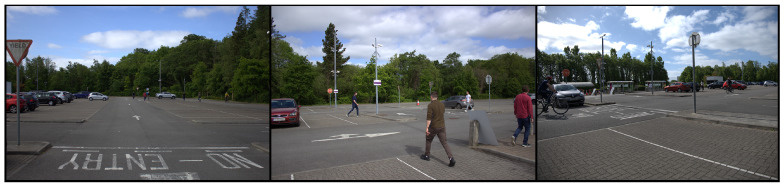
A sample image from each camera utilized in our dataset acquisition.

The raw sensor data was acquired utilizing three DSLR cameras in a controlled environment containing cars, cyclists, and pedestrians. Throughout the duration of the collection, ten subjects in total traveled along predefined paths in a car park, mimicking a generic urban scenario. These paths were changed multiple times and the subjects changed clothing throughout to increase dataset variability. Some sample images of our dataset collection can be seen in Figure 2.

The lighting varied significantly during the acquisition, with clouds intermittently covering the sun. The maximum recorded illuminance of the environment was 74 klx and the minimum was 17.5 klx. The maximum recorded luminance of an object was 5000 cd/m${}^{2}$ and the minimum was 250 cd/m${}^{2}$. The lighting was sufficient at all times for these cameras, meaning that low light is not a consideration of this study and will need further investigation in future works.

Two of the cameras were Canon 600Ds and the other was a Lumix S1. One Canon 600D and the Lumix S1 were set up on tripods and the other Canon 600D was mounted on a moving vehicle. The cameras were set up to capture a sequence of raw photos at 1 Hz. The ISO of each camera was set to 100 to minimize noise and each lens was set to an aperture of f/4. The Canon 600D has an 18MP APS-C sensor and, in this setup, had an angular horizontal field of view (FOV) of 63.3${}^{\circ }$. The Lumix S1 has a 24.2 MP full-frame sensor, and, in this setup, had an angular horizontal FOV of 73.7${}^{\circ }$. Our dataset is analogous to a front-medium FOV camera on a vehicle in a generic urban scenario.

The maximum acquisition rate of raw images for each camera was 1 Hz, or one frame per second, which limited the number of images that could be gathered. This lower frame rate has the benefit of each image being significantly different from the previous, a trait that is ideal for validating object detection, as is the case in this study. The raw sensor data were obtained from the manufacturer-specific file types, CR2 for Canon and RW2 for Lumix, using LibRaw, a library for reading raw files. The final dataset contains 580 raw images taken from the overall data collection. These images have been annotated, at full resolution, using CVAT [25] after being processed with a default ISP. The total number of car, person, and bicycle objects present in the dataset is 8156, as seen in Table 1.

#### 3.1.2. Dataset Comparison

**Figure 3:**
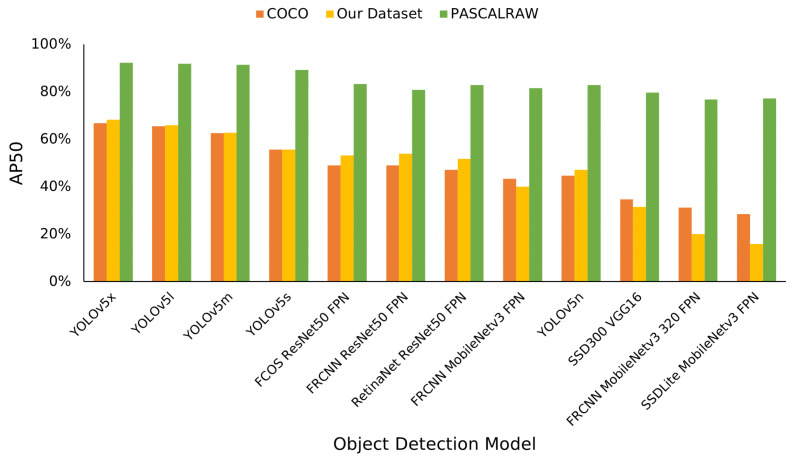
AP50 of each object detection model on our dataset, the COCO validation dataset, and the PASCALRAW dataset.

The dataset collected for this study contains, on average, 14 object instances per image, a significant increase over PASCALRAW, which contains, on average, 1.53 objects per image. The density of objects in our dataset is more similar to popular object detection benchmarks such as COCO and the Berkely Deep Drive (BDD) dataset [8] at 7.3 and 21.4 average objects per image, respectively. The object detection performance of our dataset closely matches that of COCO; however, the performance of the models on PASCALRAW is much higher due to it being a relatively easier dataset, as seen in Figure 3. The similarity of our dataset to COCO and BDD compared to PASCALRAW leads us to utilize our dataset for the remainder of this study as it is more representative of an ADAS dataset, has an object density and area distribution that more closely matches that of COCO and BDD, and has more annotated object instances as seen in Table 1.

### 3.2. Object Detection Model Selection

This study investigates 14 object detection models, outlined in Table 2. The goal of this selection was to achieve a good representation of the current state-of-the-art object detection field. All algorithms were trained on the COCO dataset for 300 epochs, except RT-DETR, which was trained for 72 epochs. The detection models were sourced from the Ultralytics GitHub [26] and the PyTorch library [27], with default parameters. The average precision (AP) achieved by the models on the COCO validation dataset, PASCALRAW dataset, and our dataset can be seen in Figure 3. As can be seen, the performance of the algorithms on our dataset is very similar to the performance on the COCO dataset, but the PASCALRAW performance is much higher, due to the larger objects and lower density of objects per image in the PASCALRAW dataset than in the others. The models utilized in this study are Faster RCNN with multiple backbones—ResNet50 FPN, MobileNetv3 FPN, and MobileNetv3 320 FPN, FCOS with a ResNet50 FPN backbone, RetinaNet with a ResNet50 FPN backbone, SSDLite with a MobileNetv3 FPN backbone, SSD300 with a VGG16 backbone, all five versions of YOLOv5 with varying numbers of parameters, the newest version of YOLO, YOLOv8, and RT-DETR, as seen in Table 2. Interpreting and correlating the results of this evaluation requires some background on the development of each algorithm and each algorithm’s key differentiator.

In 2014 Girshick et al. [28] created Regions with CNN features (RCNN). RCNN was the first object detection architecture to utilize CNNs, but it also shared design cues from traditional object detectors because it only used CNNs to generate features. Firstly, Selective Search [29] is used to produce category-independent region proposals. These proposals are fed into a large feature-extracting CNN. Lastly, these features are fed into class-specific linear SVMs to determine the class labels for these regions. RCNN development was continued in Fast RCNN [30] and Faster RCNN [31], with each bringing iterative improvements over the previous. Faster RCNN, which is evaluated in this study, swaps out the original Selective Search algorithm for a Region Proposal Network (RPN), a CNN-based method for obtaining bounding box coordinates.

The YOLO architecture was created by Redmond et al. in 2016 [32]. YOLO was the first prominent architecture to use a single-stage design. Before this, object detection was a two-stage problem: localization and classification. A single CNN unified these problems, so the whole input image is used to predict bounding boxes and class labels in a single pass. Single-stage has the added benefit of having more context for the class labels due to it being able to utilize the image area surrounding the proposed bounding boxes. Moving to a single stage with YOLO increased the inference speed while delivering a slightly lower performance than two-stage algorithms such as Faster RCNN [32]. The development of YOLO has been carried forward by multiple parties, increasing the performance and speed [22,26,33,34,35,36], and for this study, we utilize YOLOv5 [26] and YOLOv8 [36]. There are five different versions of YOLOv5 with differing numbers of parameters, from 1.9 M to 86.7 M, made for different computing capabilities. Each version is used in this study to investigate the effect of a different ISP configuration against the same architecture, but with a different number of parameters. While YOLOv5 makes use of anchor boxes in its detection head, the latest version, YOLOv8, utilizes an anchor-free approach which improves accuracy on datasets with irregularly shaped objects and speeds up the non-maximum suppression post-processing step. In this study, we include YOLOv8m to evaluate whether its improvements increase robustness to changes in the ISP.

The Single Shot MultiBox Detector (SSD), created by Liu et al. [37] in 2016, is another example of a single-stage detector. Created to run in real-time with a state-of-the-art performance, it utilizes a pre-trained image classification network backbone such as VGG16 or ResNet, similar to Faster RCNN. The backbone in SSD is utilized as a feature extractor and the feature maps are then passed into further convolutional layers that output the final bounding boxes and associated class labels at multiple scales. This multi-scale aspect allowed SSD to greatly outperform YOLO in smaller object detection; however, multi-scale feature extraction was added to YOLOv3 [34] to close this gap.

Lin et al. [38], in 2018, investigated why single-stage detectors could not achieve the same level of performance as two-stage detectors and identified foreground–background class imbalance during training as the main reason. To mitigate this, Focal Loss was created and utilized in their single-stage algorithm RetinaNet to achieve a state-of-the-art COCO AP performance. Focal Loss down-weights the higher confidence predictions to focus on the harder detections throughout training. RetinaNet uses a Feature Pyramid Network (FPN) [39] with a ResNet architecture. This FPN and ResNet backbone combination is also utilized in this study with a Faster RCNN model and the Fully Convolutional One-Stage Object Detection (FCOS) [40] model. Similar to the multi-scale feature extraction in the SSD algorithm, FPNs allow object recognition at multiple scales. SSD only utilizes upper layers for object detection, losing the higher resolution bottom layers necessary to detect small objects. FPNs make use of a top–down pathway as well as lateral connections to achieve multi-scale high-resolution feature extraction that, when paired with ResNet, achieves state-of-the-art results.

Another object detection algorithm that does not utilize anchor boxes is the Real-Time Detection Transformer (RT-DETR) [41]. RT-DETR is an end-to-end transformer-based object detection algorithm introduced in 2023 by Baidu as a follow up from the original DETR transformer-based architecture [42] that was created by Facebook AI Research. The architecture is a two-stage design, as the image is initially passed through a CNN backbone, in this case, the HGNetv2 backbone from PaddleClas [43], to generate a feature map. The feature map is then passed through a set of transformer encoder layers that use self-attention mechanisms to encode the spatial information of the image. The transformer decoder takes the feature map and the object queries as inputs and generates a set of bounding box predictions and class probabilities for each object query.

### 3.3. Software ISP

In this study, an open-source software ISP, called openISP [11,12], is utilized. This ISP implementation contains a standard set of ISP blocks, as in Figure 1 with the additional benefit of transparency to view the underlying algorithms within these blocks. Utilizing a software ISP for this study allows for a controllable and flexible ISP pipeline. Hardware ISPs generally provide minimal information to the users and only provide a set of registers to tune the camera systems. With this open-source software ISP, the full ISP implementation is available to view, making this study repeatable to test future object detection algorithms or datasets. Completing this analysis with an open-source software ISP instead of a hardware ISP allows us to control the steps by which each ISP is incremented to fully characterize the performance response of the object detection models.

The default configuration for this ISP, provided with openISP, was subjectively tuned for optimal human perception by an ISP expert for the Canon 600D and Lumix S1 individually. The green ISP blocks, as seen in Figure 1, were chosen to be investigated for this study: Color filter array (CFA) interpolation, gamma correction, bilateral noise filtering (BNF), local tone mapping, edge enhancement (EEH), hue, saturation, and contrast. While there are other denoising ISP blocks, BNF was chosen as a representative denoising algorithm. Anti-aliasing filters [44] are often used to remove color moiré; however, no such aliasing artifacts were identified in the dataset, so this ISP block was not included in the investigation. A grid search of parameters for the aforementioned chosen ISP blocks was selected and is shown in Table 3, ranging from smaller increments directly surrounding the default ISP configuration to larger increments at the extremes of each parameter. The algorithms within the chosen ISP blocks and the parameters associated with these algorithms are discussed below.

#### 3.3.1. CFA Interpolation

CMOS image sensors consist of an array of photodiodes to measure the light that the lens has focused onto the sensor. In RGB sensors, each photodiode has a filter that only allows through a given spectral band of light relating to red, green, or blue, enabling the sensor to produce color images. The photodiode filters are generally arranged in a grid pattern with twice as many green than red and blue, but other filter arrangements and spectral bands can also be utilized in these sensors. Consequently, individual photodiodes only measure the light intensity of a single spectral band, so the intensity of the other bands needs to be estimated based on the surrounding photodiode measurements. This task is known as CFA interpolation. Additionally, openISP contains two algorithms: bilinear and Malvar [45]. OpenCV’s implementation of nearest neighbor and edge-aware CFA interpolation algorithms have also been utilized in this study [45,46].

#### 3.3.2. Gamma Correction

Within the standard operating range of typical CMOS sensors, there is a linear relationship between the number of photons received at the photodiode and the raw sensor values. Human perception, however, has a non-linear relationship with the incoming number of photons. To best visualize images for human vision, gamma correction is applied via the ISP, which converts the raw photodiode values, corresponding linearly to the number of photons received, into pixel values that correspond non-linearly to the number of photons. The gamma curve responsible for the non-linear mapping is defined by a gamma parameter generally set to 0.45 [47,48] to align with human perception. In this study, the gamma has been varied from between 0.1 to 2 to evaluate the robustness of object detection models deviating from the standard gamma value.

#### 3.3.3. Bilateral Noise Filtering

There are many noise sources when capturing digital images, such as photon shot noise, dark noise, quantization noise, and pixel response non-uniformity [49]. Noise filtering is carried out in the ISP to combat this [50,51,52]. Bilateral noise filtering (BNF) [50] is utilized in this study to investigate the impact of noise filtering on the object detection performance. BNF removes noise by applying a Gaussian blur kernel, but only in areas with no edges. There are three parameters associated with BNF: the Gaussian filter kernel size, sigma value (corresponding to the kernel’s standard deviation), and an intensity sigma value corresponding to the amplitude of edges that are to be preserved. A set of BNF values has been determined, ranging from no noise filtering to significant noise filtering, that results in severe image blurring, and this set will be utilized to characterize BNF’s impact on object detection models in this study.

#### 3.3.4. Local Tone Mapping

When capturing an image, the exposure time, gain, and lens aperture determine the overall image brightness. However, there may be localized areas of low contrast within an image, particularly in dark shadow and bright highlight image regions. These low-contrast shadow and highlight regions can reduce the subjective image quality. To improve the contrast of each region individually, a local tone mapping algorithm called contrast limited adaptive histogram equalization (CLAHE) [53] is utilized. CLAHE splits the image into regions, or tiles, and performs histogram equalization on each, which increases the local contrast in these tiles by remapping the pixel values to span the full possible value range (such as 0 to 255 for an 8-bit image). Neighboring tiles are then stitched together and tile boundaries are smoothed using bilinear interpolation. An issue with the predecessor of CLAHE, adaptive histogram equalization (AHE), was that if an image tile contained a predominantly constant scene, such as a blue sky, the histogram equalization would add contrast where there should not be any while amplifying noise in the region. CLAHE fixes this issue by limiting the contrast amplification with a clip limit parameter. It does this by limiting the normalized histogram to the clip limit value prior to the histogram equalization.

#### 3.3.5. Edge Enhancement

Edge enhancement (EEH) improves the perceived sharpness of the image by first identifying and then increasing the contrast directly on sharp edges. The EEH algorithm utilized in this study is unsharp mask [54]. Unsharp mask identifies high-frequency details, or edges, in the image by Gaussian blurring the image and then subtracting the blurred image, containing the low-frequency details, from the original, leaving an edge map of high-frequency details. High-frequency details below a defined threshold, the flat threshold, are removed from the edge map to reduce image noise amplification. Once filtered by the flat threshold, the remaining edge map is multiplied by a gain factor. The edge map is clipped by the delta threshold and added back to the original image. The parameters associated with unsharp mask are Gaussian filter kernel size; edge gain (which is the multiplication factor of the edge map); flat threshold (which defines a minimum threshold for the edge map, below which the edges are not amplified); preventing weaker edges and noise from being amplified; and the delta threshold (which is the minimum and maximum value that an edge can be altered by). Due to the large number of parameters associated with this block, several parameter sets have been used, varying the edge enhancement from no enhancement to a level associated with significant oversharpening, and these parameter sets can be seen in Table 3.

#### 3.3.6. Hue

Hue is the term for the pure spectrum of colors commonly referred to by color names, for example, red, orange, yellow, and blue [55]. The hue of a pixel can be represented by a single number in degrees, which corresponds to an angular position along a color wheel. The parameter, varied in this study for the hue ISP block, is a rotational offset along this color wheel. For example, if a red pixel is rotated 180${}^{\circ }$, it becomes a cyan pixel. The hue ISP block uniformly alters the colors present in the image, which is heavily utilized in deep learning data augmentation as it deters overfitting by increasing the variance of scenes in the dataset. The hue parameter has been varied between 0${}^{\circ }$ and 300${}^{\circ }$ in 60${}^{\circ }$ increments. Hue is an ISP block that generally would not be utilized in an ADAS camera system as this is generally just used for data augmentation or stylistic effect. In this study, the hue angle was altered to simulate a color cast caused by an inaccurate auto-white balancing algorithm. Adjusting the hue angle will determine how robust object detection models are to changes in color.

#### 3.3.7. Saturation

The saturation ISP block increases or decreases the perceived saturation of colors in the image. A fully saturated color contains only a narrow spectral band of light. A desaturated color is a color that has been mixed with white, leading to lower perceived vividness. The saturation ISP block allows the image to appear more vivid, which may improve the visual quality. A saturation factor of 0 corresponds to a grayscale image, as no color is left to mix with the white. A saturation factor of 256 corresponds to 1× the saturation (meaning no change), and, in this study, the saturation factor is set to a maximum value of 1024 or 4× the saturation.

#### 3.3.8. Global Contrast

There is also a global contrast adjustment ISP block which increases or decreases the spread of the pixel values from the median pixel value. Increasing the spread of the pixels from the median pixel value increases the image’s contrast by pushing bright values brighter and dark values darker, with the drawback of increasing the number of pixels that are clipped at the minimum or maximum possible pixel value between 0 and 255 for an 8-bit image. Adding global contrast can make objects appear more segmented from their background, which can be advantageous depending on the application. Contrast gain is the only parameter associated with this block and it defines how much contrast is added or removed.

### 3.4. Metrics

In the related works section, it is evident that previous works have generally focused on AP to specify the algorithm performance. AP50, the standard metric in the PASCAL VOC dataset [56], is the area under the precision and recall curve. This curve is generated by varying the confidence threshold for a set of predictions. At a lower confidence threshold, there will be fewer missed objects, fewer false negatives, but more false positives, leading to a higher recall and lower precision. The inverse is true with a higher confidence threshold; with fewer false positives, it leads to a higher precision with a lower recall. The differing precision and recall values are plotted and interpolated, and the area under the curve is AP50. The definition of a true positive in AP50 is an intersection over union (IoU) between the model’s predicted bounding box and ground truth bounding box being greater than 50%. The primary metric in the COCO dataset, AP5095, varies the IoU threshold in 5% increments from 50% to 95% and then averages the result. This adds a positive bias for more accurate bounding boxes. These metrics are the gold standard for the overall object detection performance as they encompass true positives, false positives, false negatives, algorithm confidence, and the IoU threshold.

When characterizing a change in the performance of an object detection model, it is important to look at the individual parts that make up gold standard metrics independently. To obtain a better understanding of how the object detection model errors change due to ISP variations, they are evaluated using the toolbox for identifying object detection errors (TIDE) [57]. This toolbox classifies object detection errors into six categories: class errors, localization errors, both classification and localization errors, duplicate errors, background prediction errors, and missed object errors, while also providing false positive and false negative rates. The methodology this toolbox uses to quantify class errors is as follows: it looks at the original AP5095 performance, then fixes all classification errors and recomputes the AP5095 performance to obtain a difference in AP (dAP) caused by classification errors, resulting in a dAP metric for each error type.

In safety-critical object detection applications, such as ADAS and self-driving vehicles, it is necessary to evaluate the detection performance on difficult detection instances such as smaller objects, object occlusions, adverse weather, and low light. The effect of ISP variations on these more difficult detection instances is an unexplored topic, and, in this study, we evaluate the impact of a varying ISP on smaller objects. To evaluate small objects, specifically the COCO metric APsmall, we utilized it with a custom object area of <150 px${}^{2}$, which has been scaled from <32 px${}^{2}$ due to the difference in annotation resolution.

The score value that is reported by object detection models for each prediction represents how much confidence the detector has in the prediction being correct. In this study, the average score of all predictions, with a score > 1%, is evaluated to determine whether the degradation in performance, occurring due to ISP variations, results in reduced confidence for those predictions.

### 3.5. Analysis Pipeline

**Figure 4:**
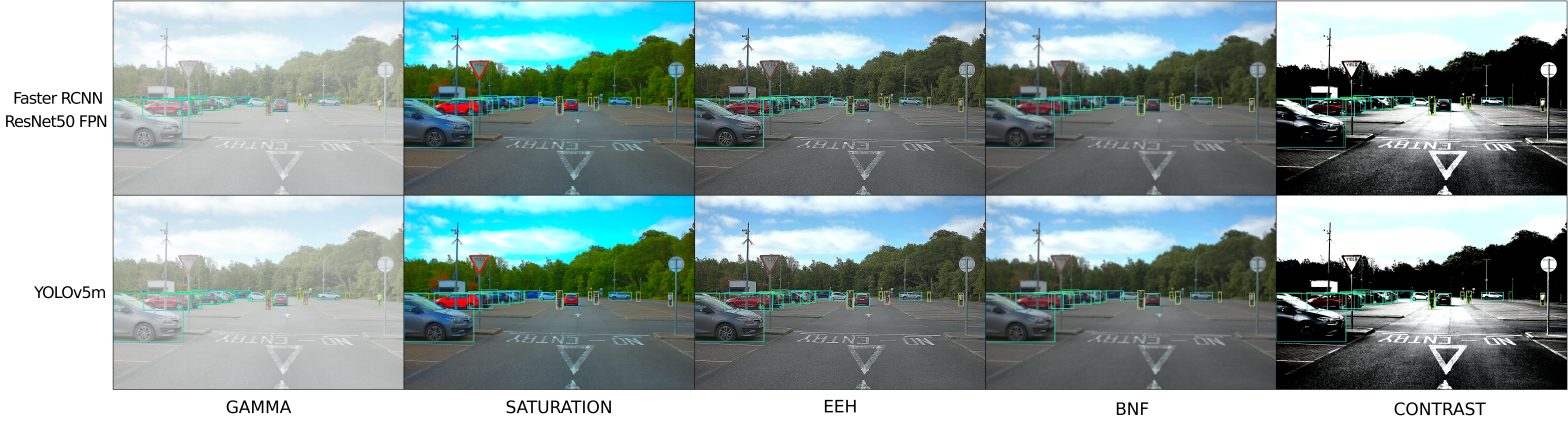
Sample detection results from YOLOv5m and Faster RCNN ResNet50 FPN across a subset of ISP configurations. Extreme ISP block configurations were chosen to depict the impact of these blocks on the image. Within the images, the green bounding boxes are ground truth, the blue boxes are detected vehicles, the orange boxes are detected people, and the pink boxes are detected bicycles.

To efficiently gather object detection performance statistics over the large search space, an automated workflow was created and is released here [58], which can be utilized to recreate the methodology within this study for other object detection models and datasets. To gather these performance statistics, first, a set of ISP parameters is taken from the grid search and the openISP configuration is updated. The raw dataset is then processed via openISP with the updated ISP configuration to produce post-ISP RGB images as seen in Figure 4. Each object detection model infers over each image and returns a set of predictions that are converted to the COCO prediction format. Each prediction set contains a single model’s predictions of all images in the dataset for one ISP configuration. These predictions are then analyzed against our ground truth using the official COCO API and the TIDE toolbox. The average confidence score of each prediction in the prediction data is also calculated for further analysis.

### 3.6. Results and Discussion

**Figure 5:**
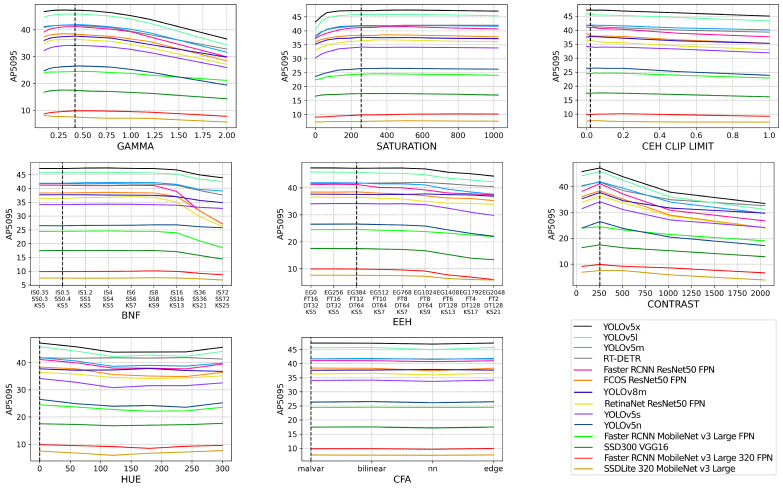
The raw dataset is processed via OpenISP [11,12] using a default ISP, however, with an altered parameter, denoted by the *x*-axis of each subplot. Each subplot refers to a different ISP block and the full set of parameters utilized can be seen in Table 3. Each of the 14 object detection models is then evaluated on each altered ISP dataset and their AP5095 performance can be seen on the *y*-axis of each subplot. The black dashed line denotes the default ISP configuration for each ISP block.

First, we look at AP5095 to review the overall object detection performance due to ISP variations in Figure 5. The different models span a wide range of the object detection performance, ranging from approx. 10% AP5095 to 50% AP5095. The best-performing algorithms among those tested are YOLOv5x, YOLOv5l, YOLOv5m, RT-DETR, and Faster RCNN with the ResNet50 FPN backbone. Figure 5 shows that there are no significant performance drops immediately surrounding each default ISP configuration, denoted by a dashed black line in each subplot, and that the performance degradation due to varying from a default ISP is a gradual one. Contrast, gamma, and saturation each cause the most significant performance degradation with smaller deviations from the default ISP configuration than the other ISP blocks. Figure 5 also proves that not all architectures have the same performance degradation characteristics due to changes in the ISP. The three algorithms utilizing a ResNet50 FPN backbone deteriorate faster when increasing the BNF. This ISP block has a blurring effect, so we can hypothesize that ResNet50 FPN backbones may be less resilient to blurring than other architectures. As expected, the performance of all YOLOv5 variants was correlated with the number of parameters in each; however, unexpectedly, YOLOv8m performs worse than YOLOv5m on our dataset. It appears that the anchor-free architecture of YOLOv8m performs worse on the smaller objects, with an APsmall of 8.2%, compared to YOLOv5m, with an APsmall of 13.8%. The performance of RT-DETR aligns very closely with YOLOv5m surrounding the default ISP, but generally retains more performance at the extreme ISP configurations. While RT-DETR is not the highest-performing model, it appears to be the most robust to changes in hue, edge enhancement, and contrast, which could be related to its transformer architecture.

**Figure 6:**
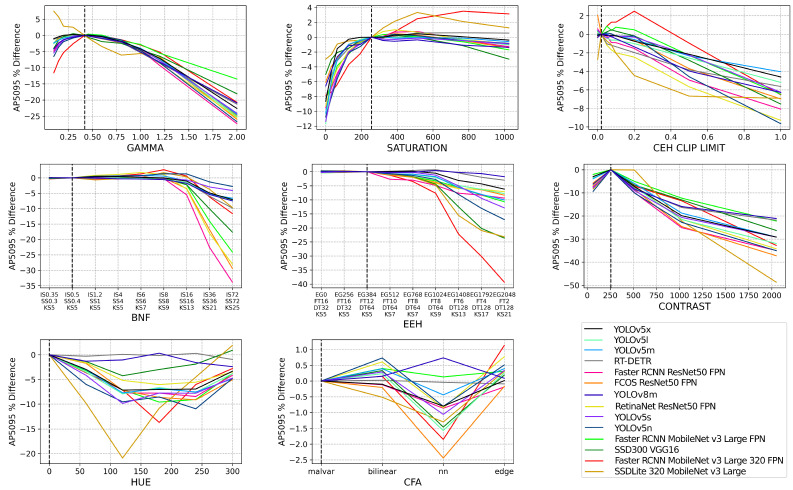
The raw dataset is processed via OpenISP [11,12] using a default ISP, however, with an altered parameter, denoted by the *x*-axis of each subplot. Each subplot refers to a different ISP block and the full set of parameters utilized can be seen in Table 3. Each of the 14 object detection models is then evaluated on each altered ISP dataset and their AP5095 performance is taken as a relative percentage difference to their AP5095 performance on the default ISP configuration dataset. The default ISP configuration is denoted by the black dashed line on each subplot.

In Figure 6, we visualize the relative percentage difference in AP5095 compared to a default ISP configuration. Where the black dashed line intersects the *x*-axis denotes the default ISP baseline configuration and the other data points are taken as relative percentage performance differences from the performance achieved on the default ISP. SSDLite320 and Faster RCNN MobileNetv3 Large 320 have a proportionally larger decrease, or in some cases, increase in performance, due to changes in the ISP as seen in Figure 6. These are the only two models that take in low-resolution images and they perform the worst overall. Both models achieve a higher performance relative to a default baseline in cases such as gamma, saturation, and clip limit, and a proportionally larger decrease in performance in EEH and hue. It is evident from Figure 6 that the performance of each model diverges when the ISP configuration is altered. The rate of performance degradation varies across each model, meaning that individual architectures have differing levels of inherent resilience to a changing ISP. Figure 6 also highlights that each ISP block causes varying amounts of performance degradation. Generalizing across all object detection models, the ordering of ISP blocks, from the highest to lowest object detection performance degradation, is as follows: contrast, EEH, BNF, gamma, hue, saturation, CEH clip limit, and CFA.

**Figure 7:**
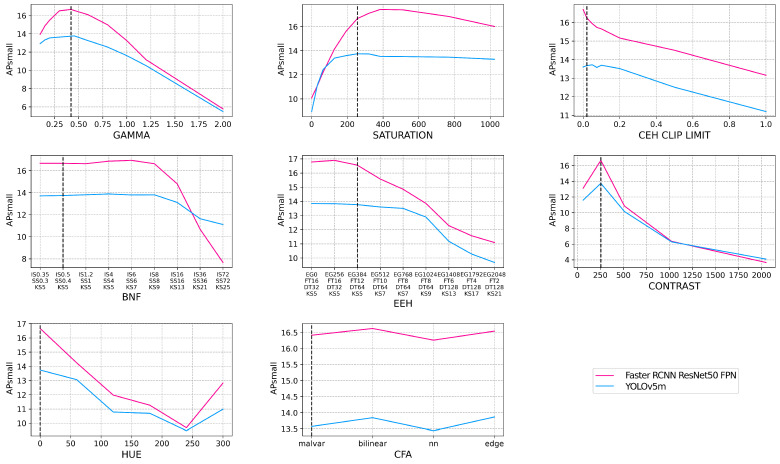
The raw dataset is processed via OpenISP [11,12] using a default ISP, however, with an altered parameter, denoted by the *x*-axis of each subplot. Each subplot refers to a different ISP block and the full set of parameters utilized can be seen in Table 3. Each of the 14 object detection models is then evaluated on the small objects (objects with a pixel area < 150 px${}^{2}$) of each altered ISP dataset and their AP5095 performance is shown on the *y*-axis as APsmall. The default ISP configuration is denoted by the black dashed line on each subplot.

In Figure 7, we take a closer look at the smaller objects for a subset of the object detection models by utilizing the COCO APsmall metric. YOLOv5m (YOLO) and Faster RCNN ResNet50 FPN (FRCNN) have been chosen for this subset as they represent single- and two-stage algorithms and achieve a similar AP5095 performance. Given that the APsmall metric was intended for the COCO dataset, with lower resolution images relative to our dataset, the APsmall threshold has been scaled accordingly from 32 px${}^{2}$ to 150 px${}^{2}$. With a default ISP applied to the dataset, FRCNN has an APsmall of 16.66%, whereas YOLO has an APsmall of 13.74%, a 2.92% difference, illustrating that FRCNN is better at detecting smaller objects. When looking at AP50, however, YOLO has an 8.87% lead over FRCNN, but FRCNN leads YOLO by 0.25% when looking at AP75, highlighting that YOLO is better than FRCNN at detecting larger objects. We can see in Figure 7 that even though FRCNN is much better when predicting smaller objects with a default ISP, it is more sensitive than YOLO when the ISP is varied as the performance of APsmall in FRCNN matches or drops below that of YOLO when varying BNF, saturation, and contrast. Figure 7 shows that varying the ISP across FRCNN and YOLO disproportionately affects smaller objects as the performance starts to degrade with less variation from the default ISP configuration, and the overall performance degradation at the extremes is higher than when looking at all objects in Figure 5.

**Figure 8:**
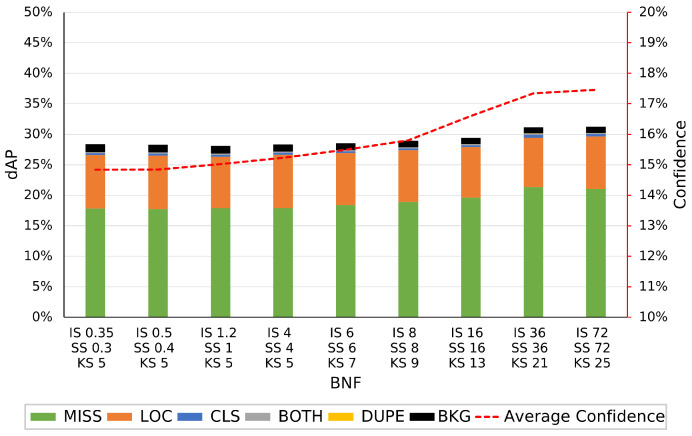
The raw dataset is processed via OpenISP [11,12] using a default ISP, however, with altered BNF parameters, denoted by the *x*-axis. The left *y*-axis shows the errors, in dAP, determined using TIDE for the YOLOv5m model. The right *y*-axis denotes the average confidence of the model over the various altered datasets.

Utilizing TIDE, we can identify what specific errors are being introduced due to the images varying from a human-viewable ISP. In Figure 8, the errors associated with YOLO on the various BNF ISP configurations are visualized. We can see that the number of missed objects is the main source of error with this object detection model for this dataset, accounting for between 17.8% and 21% dAP. Missed errors are objects that exist in the image but are not detected by the algorithm. Localization errors account for approx. 8% dAP, where these errors occur when the detected bounding box only matches the annotation box between 10% and 50% IoU, below the threshold considered to be a true positive. The remaining <5% dAP consists of background errors, class errors, combined class and localization errors, and duplicated prediction errors, in that order. As the BNF is increased, causing an increasingly blurring effect on the image, the missed error dAP increases, from 17.8% to 21%, while the other errors remain constant. The score of each YOLO prediction has been averaged across the dataset for each given ISP variation and this is visualized on the secondary axis. Visualizing the average score of the detection model indicates the model’s confidence in its predictions for a given ISP variation. When comparing this to the errors of each model, it is clear how accurate the model is at assessing its score. In Figure 8, there is a strong correlation between increasing BNF and increasing confidence. As the levels of blurring increase, the average confidence in the model’s predictions also increases. We hypothesize that due to the loss of high spatial frequency information, the model misses some of the more difficult ground truth targets, as seen in Figure 8, leaving higher overall confidence in the remaining predictions.

**Figure 9:**
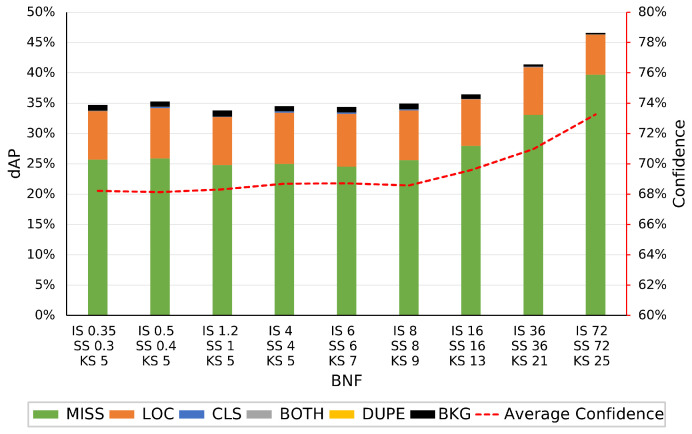
The raw dataset is processed via OpenISP [11,12] using a default ISP, however, with altered BNF parameters, denoted by the *x*-axis. The left *y*-axis shows the errors, in dAP, determined using TIDE for the Faster RCNN ResNet50 FPN model. The right *y*-axis denotes the average confidence of the model over the various altered datasets.

Figure 9 shows the error and score response of FRCNN to the various BNF configurations. The order of impact of each error type is the same as YOLO, with miss errors and localization errors making up the majority. Miss errors, in this case, vary from 24.7% to 39.7% dAP, a much larger impact than with YOLO. Localization errors remain the same as YOLO at approx. 8%. Background errors have the next highest impact at <1%, with the other error types being significantly lower. Comparing the errors associated with YOLO in Figure 8 to FRCNNN in Figure 9, we can see that while YOLO has <1% dAP in class and both type errors, FRCNN has far fewer, <0.3% dAP. FRCNN exhibits the same behavior as YOLO with an increase in missed object errors as the BNF increases. The increase in missed object errors is much greater with FRCNN and the number of localization and background errors decreases with increasing BNF, a behavior not replicated in YOLO. The average score of FRCNN, as seen in Figure 9, follows that of YOLO with an increase in confidence at higher levels of BNF. This could be due to a lower number of higher confidence, true positive predictions inferring that model confidence cannot be utilized as a metric for image quality degradation.

## 4. Data Augmentation with ISP Perturbation

In this study, we have shown that deviating from the default ISP configuration can have an impact on the performance of an object detection algorithm. In this section, we investigate if training the algorithm utilizing a range of extreme ISP configurations will make the performance of the resulting model more robust to changes in the ISP. Shorten et al. [59] provide a comprehensive review of current data augmentation techniques in which utilizing a variety of ISP configurations for data augmentation is not discussed, leading us to conclude that this is likely a novel data augmentation methodology. An object detection model robust to an ISP configuration would enable that model to be deployed on different camera systems with different ISP configurations while achieving the same performance on each. This would reduce or eliminate the current need to collect a full dataset for a single ISP configuration on a single camera system. It would also enable state-of-the-art datasets and object detection algorithms to be deployed without requiring ISP tuning.

### 4.1. Methodology

To investigate this method of data augmentation, an algorithm is trained, once on a default ISP configuration, and once with a number of ISP configurations that substantially deviate from this default configuration, as described in [58], and the performance of these models are then evaluated in the same manner as in Section 3. The ISP blocks chosen to perturb within this section are gamma, EEH, BNF, contrast, clip limit, and saturation. Faster RCNN with the ResNet50 FPN backbone has been selected as the model under test, and the dataset, processed with various ISP configurations, is utilized as the training data. The training was carried out with no other augmentations for 30 epochs and used default training hyperparameters in both cases.

### 4.2. Results

**Figure 10:**
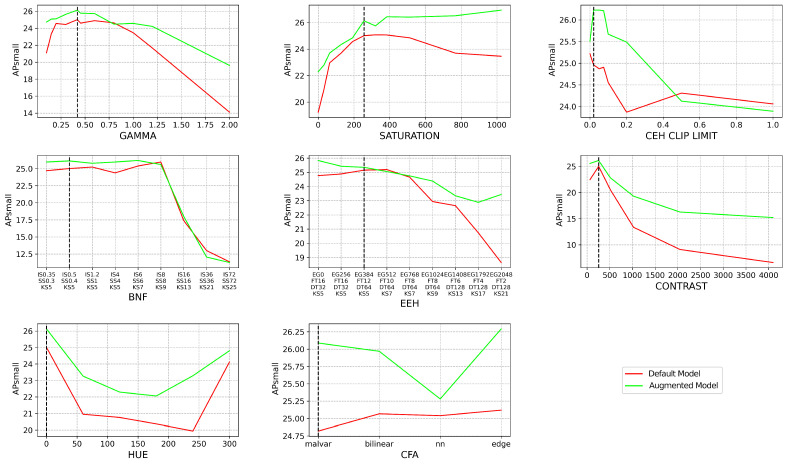
The robustness to changes in the ISP parameters is investigated with the default model, trained with an ISP, that has been tuned for human vision, and the augmented model, trained with various ISP configurations that deviate significantly from the default ISP. Both models have a Faster RCNN ResNet50 FPN architecture.

The APsmall results for both the default and augmented models can be seen in Figure 10. The APsmall metric is used as the detection of smaller objects has been shown, in Figure 7, to be more sensitive to changes in the ISP than the overall AP metric in Figure 5. Figure 10 shows that the augmented model outperforms the default model throughout the evaluation. Comparing the default to the augmented model, the augmented model has gained the most resiliency to the changing contrast, with a >5% performance gain at the most extreme contrast configuration. While the augmented model outperforms the default model in the BNF testing up to a point, both begin to degrade at the same point and degrade at the same rate. This infers that there is a limit to the blur resiliency that data augmentation can achieve and this limit sets a minimum level of sharpness needed for object detection.

## 5. Conclusions

In this study, the performance degradation of a wide range of object detection models due to varying ISP parameters has been characterized. Our results are the first to investigate the robustness of CNN-based object detection models to different ISP parameters and we have found a gradual but measurable performance decrease directly related to how much the ISP configuration differs from the default configuration. An original raw dataset and an open-source software ISP have been utilized, supporting the accuracy and repeatability of this study. By classifying the errors associated with each ISP variation, we have found that the majority of additional errors introduced when varying the ISP are missed object errors or false negatives, the least favorable errors in safety-critical applications such as ADAS or autonomous vehicles. We have found that not all CNN-based object detection architectures experience the same performance degradation due to varying ISP parameters, with the ResNet50 FPN backbone being found to suffer the most due to increasing the BNF. It was also discovered that the varying ISP parameters disproportionately affect smaller objects in the scene.

There is a need for a large-scale, publicly available raw dataset to complete this analysis by training the models before ISP analysis. Future work will adopt a similar methodology utilizing both pre-trained models and training models from scratch with a large-scale HDR ADAS dataset to fully understand the role of the ISP in-vehicle camera systems. The future large-scale dataset should also include nighttime scenes, more scene variability, and adverse weather to characterize the performance degradation of a varying ISP under these conditions. Additional ISP blocks should be investigated such as anti-aliasing, additional denoising algorithms, and HDR tone mapping. Future works should also include a multivariate analysis, in which multiple ISP blocks are altered, to characterize the impact of the object detection performance due to coupled ISP blocks.

Through data augmentation with ISP perturbation, we have found that it is possible to make CNN-based object detection models more robust to changing ISPs, allowing for pre-trained models to be deployed on camera systems with various ISPs. In our investigation of this novel data augmentation step, we have also found that there is a limit on the level of blur that can occur in an image. When increasing the BNF, both the default and augmented models suffer a very similar level of degradation at a given configuration despite the augmented model starting at a higher performance.

There is also a need to understand the effect of other sources of variation in vehicle camera systems, such as lens blurring due to manufacturing tolerances.

## Figures and Tables

**Table 1:** Number of object instances per class.

Class	# Objects	
	Our Dataset	Pascalraw
Person	2435	4077
Bicycle	604	708
Car	5117	1765
Total	8156	6550

**Table 2:** Object detection model breakdown.

Investigated Object Detection Models				
Model	Backbone	Stages	Params (M)	Year
FCOS	ResNet50 FPN	1	32.3	2019
Faster RCNN	ResNet50 FPN	2	41.8	2016
RetinaNet	ResNet50 FPN	1	34	2017
Faster RCNN	MobileNetv3 FPN	2	19.4	2019
Faster RCNN	MobileNetv3 320 FPN	2	19.4	2019
SSDLite	MobileNetv3 FPN	1	3.4	2019
SSD300	VGG16	1	35.6	2015
YOLOv5x	CSP	1	86.7	2020
YOLOv5l	CSP	1	46.5	2020
YOLOv5m	CSP	1	21.2	2020
YOLOv5s	CSP	1	7.2	2020
YOLOv5n	CSP	1	1.9	2020
YOLOv8m	CSP	1	25.9	2023
RT-DETR	HGNetv2	2	32	2023

**Table 3:** Shows the parameters associated with each ISP block alongside the minimum, default, and maximum parameter values. The default is the parameter value that is part of the default ISP configuration that was tuned for human vision.

ISP Block	Parameters	Minimum	Default	Maximum
CFA Interpolation	Malvar Bilinear Nearest Neighbor Edge-Aware	-	Malvar	-
Gamma Correction	λ Exponent	0.1	0.45	2.0
Bilateral Noise Filtering	Intensity Sigma Spatial Sigma Kernel Size	0.35 0.3 5.0	0.5 0.4 5.0	72.0 72.0 25.0
Local Tone Mapping	Clip Limit	0.0	0.02	1.0
Edge Enhancement	Edge Gain Flat Threshold Delta Threshold Kernel Size	0 16 32 5	384 12 64 5	2048 2 128 21
Hue	Hue Angle	0	0	300
Saturation	Saturation Factor	0	256	1024

## Data Availability

We have made the code used to generate these results available on GitHub [58] so that additional deep learning models and datasets can be evaluated. Unfortunately, the dataset gathered as part of this study cannot be shared due to data protection issues; however, a larger raw dataset will be made available in the GitHub repository.
